# Oxidative Stress Is Associated with Telomere Interaction Impairment and Chromatin Condensation Defects in Spermatozoa of Infertile Males

**DOI:** 10.3390/antiox10040593

**Published:** 2021-04-12

**Authors:** Benoit Berby, Cynthia Bichara, Aurélie Rives-Feraille, Fanny Jumeau, Pierre Di Pizio, Véronique Sétif, Louis Sibert, Ludovic Dumont, Chistine Rondanino, Nathalie Rives

**Affiliations:** 1 Biology of Reproduction—CECOS Laboratory, Rouen University Hospital, Normandie University, UNIROUEN, EA 4308 “Gametogenesis and Gamete Quality”, F 76000 Rouen, France; benoit.berby@gmail.com (B.B.); cbichara4@gmail.com (C.B.); aurelie.rives@chu-rouen.fr (A.R.-F.); fanny.jumeau@chu-rouen.fr (F.J.); pierre.di-pizio@chu-rouen.fr (P.D.P.); veronique.duchesne@chu-rouen.fr (V.S.); 2 Department of Urology—Andrology, Rouen University Hospital, Normandie University, UNIROUEN, EA 4308 “Gametogenesis and Gamete Quality”, F 76000 Rouen, France; louis.sibert@chu-rouen.fr; 3 Normandie University, UNIROUEN, EA 4308 “Gametogenesis and Gamete Quality”, F 76000 Rouen, France; ludovic.dumont1@univ-rouen.fr (L.D.); christine.rondanino@univ-rouen.fr (C.R.)

**Keywords:** chromatin condensation, male infertility, oxidative stress, spermatozoa, telomere

## Abstract

Telomere length can be influenced by reactive oxygen species (ROS) generated by lifestyle factors or environmental exposure. We sought to determine whether oxidative stress has an impact on sperm nuclear alterations, especially on chromatin organization and telomere interactions in the spermatozoa of infertile males. We performed an observational and prospective study including fifty-two males, allocated in the “case group” (30 infertile males presenting conventional semen parameter alterations) and the “control group” (22 males with normal conventional semen parameters). ROS detection was determined on spermatozoa using CellROX^©^ probes. Sperm nuclear damage was assessed using quantitative fluorescence in situ hybridization (Q-FISH) for relative telomere length and telomere number, aniline blue staining for chromatin condensation, terminal deoxynucleotidyl transferase dUTP nick-end labeling for DNA fragmentation, and FISH for aneuploidy and 8-hydroxy-2′-deoxyguanosine immunostaining for oxidative DNA damages. Infertile males had significantly increased levels of cytoplasmic ROS and chromatin condensation defects as well as a higher mean number of telomere signals per spermatozoon in comparison with controls. In addition, the mean number of sperm telomere signals were positively correlated with the percentage of spermatozoa with chromatin condensation defect. In infertile males with conventional semen parameter alterations, oxidative stress is associated with telomere interaction impairment and chromatin condensation defects.

## 1. Introduction

Oxidative stress, defined as a perturbation of local or systemic redox regulation, may be responsible for male infertility [1]. Reactive oxygen species (ROS), also known as free radicals, are subproducts of cellular respiration, and their over-production, accumulation, or defective elimination leads to oxidative stress [2]. In the male urogenital tract, ROS originate mainly from leukocytes and immature abnormal spermatozoa, their production is triggered by various clinical conditions such as infection or inflammation, varicocele, cryptorchidism, testicular torsion, and toxic exposure [3,4]. Due to their extremely reduced cytoplasm, spermatozoa have low amounts of antioxidant agents, notably enzymatic ones and consequently they use the high antioxidant capacity of seminal plasma [3,5].

However, in moderate concentration, ROS play a major role in the post-testicular sperm maturation. They are involved in the formation of inter-protamine disulfide bridges during epididymal transit, reinforcing the spermatozoa nuclear condensation [6]. ROS also participate in the phosphorylation of membranous tyrosine, which allows capacitation and flagellar hyperactivation [7].

Oxidative stress can be responsible for male infertility, as a relationship has been established between ROS, male infertility, and conventional semen parameter alterations. Exposition to oxidative stress can lead to DNA damages in the spermatozoa of infertile males [7,8,9,10,11]. Sperm DNA attacks by ROS are thought to be a step in the cascade reaction leading to DNA fragmentation and ultimately to apoptosis [7,12].

Some of the canonical targets of oxidative stress are telomeres [13], chromatin loops located at chromosome ends made of repeated DNA sequences (5′-TTAGGG-3′) [14,15] and covered by a sheltering associated protein complex named telosome. Telomeres participate in the maintenance of chromosomal integrity, preventing reconnaissance of molecular DNA free terminations by the cellular repairing machinery [16]. Besides their role of protecting chromosome ends from degradation and fusion, they ensure homologous pairing during cell division [17]. Telomere length varies individually and decreases after each cell division in the absence of telomerase [18]. Telomere length can be influenced by several factors such as gender, age, and ROS generated by lifestyle factors or environmental exposure [15]. Therefore, it has been well established that ROS contribute to telomere attrition [19].

In the male germ cell lineage, spermatogonia and spermatocytes I express telomerase at a very high level, which is a unique condition during adulthood. Telomeres are generally the longest in spermatozoa compared to other immature germ cells, more specifically spermatogonia [20]. Furthermore, several studies found that older men have longer sperm telomere length (STL) than younger ones [17,21,22,23]. In addition, infertile patients have a diminished STL compared to fertile controls [21,24,25,26]. Indeed, men with oligozoospermia have a significantly diminished STL compared to men with normal conventional semen parameters [27].

Few studies have investigated the link between oxidative stress and STL. Oxidative stress is commonly admitted to be a major cause of telomere shortening in somatic cells [28]. The addition of hydrogen peroxide, an effective ROS, to spermatozoa leads to a decrease of STL measured by quantitative fluorescence in situ hybridization (Q-FISH) [29]. In infertile males, ROS quantification by chemoluminescence in seminal plasma was not correlated to STL [25]. In another study, severe oxidative stress (i.e., high concentration of ROS in seminal plasma) has been associated with diminished STL [30].

Considering the association between oxidative stress, nuclear damages, and telomere attrition, and in order to deepen the relationship between these different parameters, we conduct a pilot study to explore ROS expression, oxidative DNA damages, telomere length and number, as well as nuclear quality in the spermatozoa of infertile males presenting conventional semen parameter alterations in comparison with spermatozoa of males with normal conventional semen parameters.

## 2. Materials and Methods

### 2.1. Population, Study Design, and Participants

A prospective monocentric case-control study was conducted at the Reproductive Biology Laboratory-CECOS of Rouen University Hospital from 1 January 2016 to 31 August 2016. Patients were recruited in accordance with the 1975 Declaration of Helsinki principles of human experimentation (protocol code 2020002; date of approval 30 November 2020). They were informed of the potential use of the residues of the semen collection carried out in the context of diagnosis for research purposes, and they did not express their opposition to the research in accordance with current French law. Only surplus semen was used in this study. The study was approved by the Ethical Review Board of the French CECOS network.

Clinical and biological data were collected during the pre-examination consultation: age (years), Body Mass Index (BMI) (kg/m^2^), duration of infertility (months), urological history that might affect male fertility (cryptorchidism, varicocele, testicular trauma, orchi-epididymitis), lifestyle parameters (tobacco, alcohol, or cannabis consumption) (yes/no), and professional toxic exposure (yes/no).

Patients of the case group were infertile males who presented at least oligozoos]ermia, defined according to World Health Organization (WHO) laboratory guidelines [31] as sperm concentration less than 15 × 10^6^ per mL and total sperm count less than 39 × 10^6^ spermatozoa per ejaculate and associated or not with another conventional semen parameter alteration such as progressive motility (WHO grades a + b combined, %) less than 32%, vitality (live spermatozoa, %) less than 58%, and sperm normal morphology (%)—assessed according to David’s modified classification [32]—less than 30%. Patients of the control group had normal conventional semen parameters according to the WHO criteria mentioned above and consulted for female or unexplained infertility.

Sperm nuclear quality was evaluated using terminal deoxynucleotidyltransferase-mediated dUTP nick end labelling (TUNEL) for DNA fragmentation, aniline blue (AB) staining for chromatin condensation, 8-Oxo-2′-deoxyguanosine (8-OHdG) immunostaining for DNA oxidative damages, fluorescence in situ hybridization (FISH) for chromosome aneuploidy, and peptide nucleic acid probes (PNA) for quantitative FISH (Q-FISH) to measure telomere length and count the number of telomere signals.

Semen samples were collected into a sterile container (Clinisperm^®^, CML, Nemours, France) directly at the laboratory after sexual abstinence for 3 to 5 days according to WHO quality guidelines [31]. In addition, a sample of 1 mL was divided in two aliquots: one aliquot for direct ROS assessment using CellROX^©^ probes (Life technologies, Molecular Probes, Eugene, OR, USA) on fresh spermatozoa, one aliquot for nuclear quality assessment on fixed spermatozoa.

### 2.2. Sperm Nuclear Analyses

#### 2.2.1. CellROX^©^ Staining

CellROX^©^ are vital, fluorogenic probes widely used in toxicology assays. The cell-permeable reagents are non-fluorescent in a reduced state and exhibit a strong fluorogenic signal upon oxidation. CellROX^©^ Green Reagent (C10444, Life technologies, Molecular Probes, Eugene, OR, USA) is a DNA dye that, upon oxidation, binds to DNA with a signal localized primarily in the nucleus and mitochondria. In contrast, the signal from CellROX^©^ Deep Red Reagent (C10443, Life technologies, Molecular Probes, Eugene, OR, USA) is localized in the cytoplasm.

**Figure 1:**
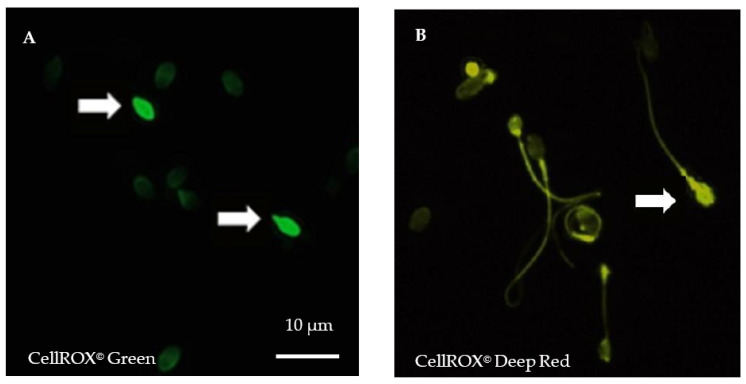
Cytoplasmic and nuclear reactive oxygen species (ROS) detection in spermatozoa after staining using CellROX^©^ fluorescent probes. (**A**). Spermatozoa staining using vital fluorogenic CellROX^©^ Green probes. (**B**). Spermatozoa staining using fluorogenic CellROX^©^ Deep Red probes. In a reduced state, CellROX^©^ probes are non-fluorescent and oxidation triggers a strong fluorescence. Positive, oxidized spermatozoa exhibit strong head fluorescence (white arrows). Midpiece or tail fluorescence is not considered positive. Magnification ×900, exposure time of 800 ms, acquisition with Bioview Duet v2.3 image analyzer (Nes Ziona, Israël).

For each semen sample, fresh spermatozoa were incubated either with Phosphate Buffer Saline (PBS) 1X or with 30% H_2_O_2_ (positive control) for 10 min at room temperature (RT) before incubation with both CellROX^©^ (1 µL of each) probes for 10 min in darkness at RT. After washing in PBS, spermatozoa were fixed in formaldehyde 3.7% (Sigma-Aldrich by Merck, Saint-Quentin Fallavier, France), before spreading and air-dried in darkness. Counterstaining was performed using 4′,6-diamidino-2-phenylindole (DAPI II, Adgenix, Voisin le Bretonneux, France) diluted in antifading mounting medium (Antifade^®^, MP-QbioGene, Illkirch, France). Images were captured at a ×900 magnification by a U-CMAD3 camera mounted on a BX61 epifluorescence microscope (Olympus, Tokyo, Japan) with a fixed acquisition and exposure time of 800 ms (Figure 1). Image acquisition and analyses were carried out using the Bioview Duet v2.3 image analyzer (Nes Ziona, Israël). For each patient, five hundred spermatozoa were examined.

#### 2.2.2. 8-OHdG Immuno-Detection

Oxidative damages to DNA were assessed by 8-OHdG immunostaining, as adapted from previous work [33]. Spermatozoa were fixed with 4% (*w/v*) paraformaldehyde (Sigma-Aldrich by Merck, Saint-Quentin Fallavier, France) for 20 min before spreading onto a glass slide and DNA decondensation using 1 M NaOH for 2 min. Non-specific binding sites were blocked with 5% (*w/v*) bovine serum albumin (BSA in PBS, Sigma-Aldrich by Merck, Saint-Quentin Fallavier, France) for 30 min at room temperature and then rinsed twice again. Slides were incubated overnight at 4°C with mouse monoclonal anti-8-OHdG IgGs (2 μg/mL in PBS + 5% (*w/v*) BSA, clone 15A3, NB110-96878, Novus Biologicals, Littleton, CO, USA). Slides were then rinsed three times in PBS with Tween 20% (Sigma-Aldrich by Merck, Saint-Quentin Fallavier, France) and then incubated 1 h at 4 °C with an anti-Fc IgG coupled to Alexa 488 fluorochrome (#ab150077; Abcam, Cambridge, MA). Slides were counterstained with propidium iodide (Counterstain C2, Cambio, Cambridge, United Kingdom) diluted 1:1000 in antifading mounting medium (Antifade^®^, MP-QbioGene, Illkirch, France). A total of 500 spermatozoa were counted under an epifluorescence microscope (Leitz DMRD^®^, Leica, Solms, Germany). A positive control was used by exposing a random semen sample to 30% H_2_O_2_ 10 min at RT prior to fixation in paraformaldehyde. Sperm nuclei were considered positive and to present oxidative DNA damages, if more than 50% of the head area showed a green fluorescence. The percentage of spermatozoa with oxidative DNA damages was calculated per patient by the ratio between the number of sperm nuclei positive for 8-OHdG immuno-detection and the total number of explored spermatozoa.

#### 2.2.3. TUNEL Assay

After fixation with methanol for 30 min at −20 °C, spermatozoa were spread onto glass slides and stored at −20 °C until TUNEL assay to analyze DNA fragmentation using the In situ Cell Death Detection^®^ kit (Roche, Mannheim, Germany) according to the manufacturer’s instructions and as previously described (34). DNA fragmentation was characterized on 500 spermatozoa at a ×1000 magnification using an epifluorescence microscope (DMRB^®^, Leica, Solms, Germany). Spermatozoa were considered to have fragmented DNA when the green fluorescence signal occupied more than 50% of the head area. The percentage of spermatozoa with DNA fragmentation was calculated per patient by the ratio between the number of sperm nuclei positive for TUNEL assay and the total number of explored spermatozoa.

#### 2.2.4. Aniline Blue Staining

Sperm chromatin condensation was evaluated by AB staining at 5% pH 3.5 for 5 min (Gurr^®^ by VWR™ Chemicals BDH^®^, Fontenay-sous-Bois, France), as previously described [34]. Five hundred spermatozoa were analyzed at a ×1000 magnification under a light microscope (Leitz DMRD^®^, Leica, Solms, Germany). Sperm nuclei were considered positive for AB staining and to contain decondensed chromatin when the dark blue staining occupied more than 50% of the head area. The percentage of spermatozoa with abnormal chromatin condensation was calculated per patient by the ratio between the number of sperm nuclei positive for AB staining and the total number of explored spermatozoa.

#### 2.2.5. FISH Assay

A three-color fluorescence in situ hybridization (FISH) was performed to assess the frequency of chromosomal abnormalities, as previously reported [34] using α-satellite centromeric probes specific for chromosomes 18 (CEP 18 Spectrum Aqua Blue™, Abbott, Rungis, France), X (CEP X Spectrum Green™, Abbott, Rungis, France) and Y (CEP Y Sat III Spectrum Orange™, Abbott, Rungis, France). A minimum of 5000 sperm nuclei was examined at a ×1000 magnification using a BX61 epifluorescence microscope (Olympus, Tokyo, Japan), according to criteria previously described [34]. The rate of diploidy was calculated as the sum of rates of presumed XX, YY, and XY diploid spermatozoa. The rate of aneuploidy was calculated as the sum of rates of presumed disomic XX, YY, 1818, and hyperhaploid XY spermatozoa. The rate of total chromosome abnormalities was considered as the sum of the rates of aneuploidy and diploidy.

#### 2.2.6. Quantitative FISH (Q-FISH) for Telomere Assessment

Telomere relative length and number were determined by Q-FISH using peptide nucleic acid (PNA) probes as previously described [33,35,36]. After fixation in methanol, sperm nuclei were decondensed with 1M NaOH for 3 min before fixation with 3.7% (*w/v*) formaldehyde (Sigma-Aldrich by Merck). The Telomere PNA FISH kit (Dako, K5326, Les Ulis, France) was used following manufacturer’s instructions (DakoCytomation). Cy3-labelled (532 nm, red fluorescent signal) PNA telomere probes were mixed with FITC-labelled (488 nm, green fluorescent signal) PNA centromere of chromosome 2 probe, kindly provided by DakoCytomation, Denmark. The mix was spread onto the slide then denatured at 80 °C for 5 min and hybridized at 32 °C for 2 h using Thermobrite (Abbott Molecular, Abbott Park, IL, USA). Slides were counterstained with 4′,6-diamidino-2-phenylindole (DAPI II, Adgenix) diluted in antifading mounting medium (Antifade^®^, MP-QbioGene, Illkirch, France). Images were captured at a ×900 magnification on a BX61 epifluorescence microscope (Olympus, Tokyo, Japan). Ten consecutive images at different focal depths were stacked into a composite image used for numeration. Image acquisitions and analyses were carried out using the Bioview Duet v2.3 image analyzer (Biovie, Nes Ziona, Israël). A manual analysis of spermatozoa heads was performed in order to assess the number of telomere signals present in each nucleus. Only spermatozoa with exactly one green spot were taken in account and any other configuration has been excluded. The fluorescence intensity of fluorescent signals was automatically analyzed after correction for background autofluorescence, exposure and acquisition times with a specifically designed program for telomere length developed by the manufacturer (Biovie, Nes Ziona, Israël). Chromosome 2 centromere probe intensity was used to normalize the signal leading to a relative telomere length, expressed in Fluorescent Relative Unit (FRU): (telomere probe intensity)/(centromere 2 probe intensity) ×100. A total of 200 spermatozoa was assessed for each sample.

### 2.3. Statistical Analyses

Statistical analysis was performed using R 3.2.2 (R Foundation for Statistical Computing, Vienna, Austria). Data were described using the following descriptive parameters: mean and standard deviation (s.d) for quantitative variables and frequencies for qualitative variables. The conventional semen parameters and sperm nuclear alterations were expressed per case and control groups. The case male population was compared to the control male population using χ^2^ test for qualitative variables and non-parametric Mann-Whitney tests for quantitative variables. The Spearman rank correlation coefficient (*r*) was calculated to assess the correlation between the different sperm nuclear alterations. A *p*-value less than 0.05 was considered significant.

## 3. Results

### 3.1. Population Description

A total of 52 individuals were included in the study, allocated in two groups: a control group of 22 males aged between 21 and 53 years and a case group of 30 infertile males aged between 23 to 45 years (Table 1). In the control group, 54.5% (*n* = 12) and 45.5% (*n* = 10) had respectively fathered previously or faced unexplained infertility. Urological histories, BMI, rates of professional toxic exposure, as well as alcohol, tobacco, and marijuana use were similar between the two groups. The female infertility factor, such as tubal infertility, dysovulation, endometriosis or a low ovarian reserve did not differ significantly between the two groups. In our control group, normozoospermic males with unexplained infertility did not differ significantly in any features compared to normozoospermic males who had previously conceived spontaneously (Supplementary Table S1).

All males of the control group presented normal conventional semen parameters (Table 2). In the case group, 50% (15 out of 30) of males had an isolated oligozoospermia, 16.7% (5 out of 30) an oligoasthenozoospermia, 13.3% (4 out of 30) an oligoteratozoospermia and 20.0% (6 out of 30) an oligoasthenoteratozoospermia. No difference was found between the two groups regarding sexual abstinence, semen volume, and leukocytospermia.

### 3.2. Sperm Nuclear Abnormalities

Sperm nuclear abnormalities are presented in Table 3. The rate of spermatozoa positively stained with CellROX^©^ DeepRed probe was significantly higher in the case group (*p* = 0.039), while no difference was found with CellROX^©^ Green probe (*p* = 0.66). The percentage of 8-OHdG positive spermatozoa did not vary significantly between the two groups (*p* = 0.7). Chromatin condensation defects were significantly higher in the case group (*p* = 0.006). The rate of sperm DNA fragmentation was comparable between the two groups (*p* = 0.49), as well as sperm aneuploidy (*p* = 0.28), diploidy (*p* = 0.99), and total chromosome abnormalities (*p* = 0.70). The mean number of telomere signals by spermatozoa was significantly higher in the case group (*p* = 0.049), while sperm relative telomere length did not vary between the two groups.

**Figure 2:**
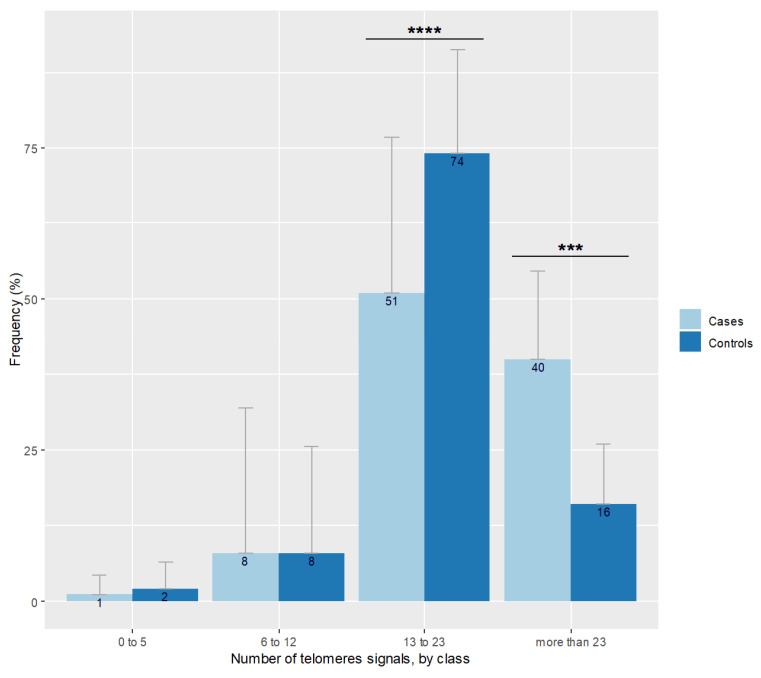
Number of telomere signals represented by class observed in spermatozoa of cases and controls. Bar plot represents the frequency of telomere signal number visualized in individual spermatozoon, classed with threshold at 5, 12, and 23 signals. Error bars show standard deviations. Cases are in pale blue and have more spermatozoa with more than 23 signals (*** *p* = 0.001), and less with 13 to 23 signals (**** *p* < 0.001) than controls represented in dark blue.

A moderate positive correlation was detected between sperm the DNA fragmentation rate and the percentage of 8-OHdG positive spermatozoa (r = 0.29, *p* = 0.042). The number of sperm telomere signals increased with sperm chromatin condensation defects (r = 0.29; *p* = 0.04). Neither the number of telomeric signals nor sperm chromatin condensation defects were correlated to CellROX DeepRed^©^ staining (*p* = 0.21 and *p* = 0.27 respectively). No correlation was found between patient age and the number of telomere signals (*p* = 0.82) nor with telomere length (*p* = 0.41). Sperm relative telomere length decreased when the mean number of telomere signals grew (r = −0.30; *p* = 0.035). Figure 2 shows the repartition of the number of telomeric signals in the case group. The distribution differed according to the percentage of spermatozoa expressing a number of telomere signals comprised between 13 and 23 in favor of controls (*p* < 0.001) and in favor of cases regarding the more than 23 signals class (*p* = 0.001).

## 4. Discussion

Our pilot study highlighted a significant increase of cytoplasmic ROS and chromatin condensation defects in sperm nuclei of infertile males with oligozoospermia alone or associated with other semen parameter alterations. We also showed a significant increase of the number of telomere signals in sperm nuclei of infertile males compared to the normozoospermic control group. Among the case group, the number of telomere signals increased significantly with sperm chromatin condensation defects. 

Our data showed an increase of cytoplasmic ROS but no variation of nuclear ROS, oxidative damage to DNA (8-OHdG immunostaining), DNA fragmentation, and telomere length in spermatozoa of infertile males. Oligozoospermic males are known to present higher level of seminal fluid ROS concentration [9,37,38]. Seminal fluid measurements of ROS concentration or total antioxidant capacity are valuable tools but do not reach a cellular level of precision [3]. In the current study, the CellROX^©^ assay used to determine the presence of ROS present in spermatozoa allows a more precise evaluation than indirect assessment in seminal fluid [39]. Indeed, it makes it possible to study each spermatozoon individually and have been previously used in animal spermatozoa [39,40,41]. CellROX^©^ probes are emerging as a marker of interest in human andrology. To the best of our knowledge, our study is the first to apply such a methodology to human spermatozoa of infertile men in combination with the assessment of several sperm nuclear alterations. A recent study using CellROX^©^ Orange probes showed their location in sperm midpiece and sensitive to H_2_O_2_ rather than to superoxide [42]. Moreover, the mean fluorescence intensity assessed via flow cytometry and the percentage of labeled cells assessed via fluorescence microscopy were comparable for this specific probe, while they differed for other fluorescent probes [39].

We chose to assess STL by Q-PNA-FISH with epifluorescence microscopy. In opposition to the widely used qPCR assays, it limits us to a relative measurement but allows to count telomere signals and measure telomere length in individual spermatozoon [21,28]. This method has already been a major contributor to sperm nucleus biology since the first studies showing the feasibility of centromeric-telomeric Q-FISH to assess telomere length [43]. Most of studies regarding STL are performed using qPCR which is a reliable, high throughput method [22,23,25,30]. The main inconvenience of this procedure is to deliver a mean relative STL according to a household gene for a whole sperm sample, while PNA Q-FISH allows STL measurement of each spermatozoa separately. Furthermore, qPCR leads to variability between the different studies, especially due to the quality of DNA extraction form spermatozoa [44,45]. Absolute quantification can be performed by Southern blotting of terminal restriction fragment (TRF) [24], or using telomeric probes for flow-FISH [21] or for Q-FISH by comparing STL of the explored cell sample to a known telomere length cell linage such as murine lymphoma’s L-2178-S [17]. Without this control, Q-FISH results only in a relative measurement of TL. 

To the best of our knowledge, only three previous studies used the same methodology to measure STL as the one proposed by our research team. The first study compared STL between 10 semen samples with high sperm DNA fragmentation level detected after acridine orange staining and 10 semen samples with low sperm DNA fragmentation level showing an increase of telomere number in the group of patients with high sperm DNA fragmentation level [28]. The second one performed on 45 unselected infertile males did not find any association between sperm DNA fragmentation level and telomere length but showed a negative correlation between sperm DNA fragmentation level and the mean number of sperm telomeres [17]. These data were not observed in our study and Moskovtsev’s one [28]. In addition, in most of qPCR studies, a positive correlation was reported between STL and male age [22]. No correlation was detected between STL and DNA fragmentation in spermatozoa of 42 infertile males. In addition, patients achieving pregnancy have longer telomeres than those who did not [29]. We only observed a moderate positive correlation between sperm DNA fragmentation rate and the percentage of 8-OHdG positive spermatozoa (r = 0.29, *p* = 0.042). As generally proposed, sperm DNA fragmentation may be due to several reasons including a pro-oxidative situation, such as unrepaired meiotic breaks, poor evacuation of apoptotic germ cells during spermatogenesis or/and necrotic cells during epididymal maturation, mechanical breaks in the sperm nucleus upon spermiogenesis during the replacement of histones by protamines [46].

A recent study confirmed the non-random organization of sperm chromatin using a 3D PNA FISH assessed in normozoospermic males [47]. Telomeres were preferentially located in clusters into the peripherical and intermediate radial regions and in the mid longitudinal part of nucleus rather than into interior region and head or tail parts. Ioannou et al. reported the formation of more than one chromocenter (i.e., cluster of centromeres) in a patterned scheme [47]. This study contradicts the commonly accepted model where a unique interior chromocenter radiates chromosomes with telomeres anchored exclusively to peripherical region and enforced a more segmented organization of chromosomes. This study reported that 2D and 3D assessment of PNA Q-FISH smears correlate but 3D methods ensure a greater number of telomere signals. The acquisition and assessment of nine stacked images for each nucleus helped us to prevent such a bias, which is supported by our very similar mean number of telomere signals compared to the study of Ioannou et al. (20.47 vs. 20.77, respectively) [47].

We observed an increase of chromatin condensation defects among infertile males with oligozoospermia, in agreement with previously published data [34,48,49,50]. Oxidative stress is responsible for defects of chromatin condensation, in sperm or somatic cells. It affects sperm chromatin packaging by perturbing the non-random ordering of mammalian chromosome in sperm nucleus [51]. Defects of chromatin condensation led to an increase of telomere signals in nucleus, due to the perturbation of chromatin architecture. In the sperm nucleus, telomeres are organized in dimer and tetramer anchored in the nuclear membrane [14,20]. Former works explored the telomeres dimers in sperm, showing that *p* and q telomeres are spatially adjacent, and thus telomere dimers represent the associated ends of each chromosome [14]. 

Disruption of chromatin homeostasis and detachment of telomere polymers from nuclear membrane might be responsible for chromosome disorganization in the sperm nucleus [51,52]. Furthermore, STL have been positively associated with protamination of human sperm [23], leading to the hypothesis that shorter telomeres are more susceptible of disruption. This is emphasized by our finding that the mean number of telomere signals increased when the relative telomere length decrease. Even if in vitro exposure to an oxidative agent, H_2_O_2_, shortened the STL measured by PNA Q-FISH [29], we did not observe this shortening in our study.

Our study has several limitations. The number of participants in the control group was limited and only 54.5% and 45.5% had respectively fathered previously or faced unexplained infertility. Our sample size was not sufficient to consider the potential effect of life style factors or professional exposure on meiotic disturbances. Indeed, short telomeres could be linked to abnormal semen parameters, such as low sperm count, reduced motility, and increased DNA fragmentation [53]. Obesity is a well-known factor in male infertility and is associated with STL shortening [54]. A recent study failed to link sperm telomere length to lifestyle factors such as caffeine and alcohol consumption, exercise, and smoking [55]. The value of STL as a predictor of the success of Assisted Reproductive Techniques is disputed by experts in the field [15]. Indeed, no pregnancies were achieved after in vitro fertilization or Intracytoplasmic Sperm Injection (ICSI) using spermatozoa presenting atypical STL, exceptionally large or unusually small ones [27], whereas STL measurement was not considered to be useful to predict reproductive outcomes in ICSI cycles using donor semen [56]. In addition, at the time of the conceptualization of the present study, the Male Oxidative Stress Infertility (MOSI) concept was not formally defined and originated afterwards by Agarwal et al. [57]. Therefore, we were unable to take into account this aspect in our study. However, in our control group, normozoospermic males with unexplained infertility were comparable to normozoospermic males who had previously conceived spontaneously.

## 5. Conclusions

This is the first study showing that the number of telomere signals, chromatin condensation defects, and ROS expression in sperm cytoplasm were significantly higher in spermatozoa of infertile males with semen parameter alterations. This consideration could be used as an argument in the debate over the use of antioxidants in infertile men. Antioxidant therapeutics, such as combination of vitamins C and E, selenium, and N-acetylcysteine have a controversial effect on chromatin [58]. Obviously, the present work needs confirmation in a larger population of infertile males with semen parameter alterations.

## Figures and Tables

**Table 1:** Clinical parameters in case and control groups.

Clinical Parameters	Case Group *n* = 30		Control Group *n* = 22		*p*
**Age**	35.23	±8.04	35.09	±5.71	0.81
years; mean. ± s.d					
**Body Mass Index (BMI)**	26.45	±5.39	24.87	±2.69	0.44
kg/m^2^; mean. ± s.d					
**Duration of infertility**	42.3	±42.7	38.3	±23.8	0.73
months; mean. ± s.d					
**Urological history**	10	33.3	6	27.3	0.87
yes; (*n*, %)					
**Cryptorchidism**	6	20.0	3	13.6	
**Varicocele**	2	6.7	3	13.6	
**Testicular trauma**	2	6.7	0	0.0	
**Orchi-epididymitis**	0	0.0	0	0.0	
**Professional toxic exposure**	8	26.7	5	22.7	1.0
yes; (*n*, %)					
**Tobacco consumption**	15	50.0	12	54.5	0.97
yes; (*n*, %)					
**Alcohol consumption**	23	76.7	19	86.3	0.60
yes; (*n*, %)					
**Marijuana consumption**	6	20	1	4.5	0.23
yes; (*n*, %)					

s.d: standard deviation; *n*: number; %: percent.

**Table 2:** Conventional semen parameters in case and control groups.

Semen Parameters	Case Group		Control Group		*p*
	mean ± s.d				
**Sexual abstinence**	5.3	±2.6	5.6	±2.1	0.47
(days)					
**Volume**	4.6	±1.1	4.4	±1.9	0.35
(mL)					
**Sperm count**	6.8	±2.5	47.7	±20.7	**9 × 10^−8^**
(10^6^/mL)					
**Total sperm number**	30.3	±10.7	197.6	±102.0	**9 × 10^−8^**
(10^6^/ejaculate)					
**Sperm progressive motility**	33.5	±8.6	40.5	±5.5	**0.003**
(a + b, %)					
**Vitality**	74.8	±10.4	79.8	±6.4	0.09
(live spermatozoa, %)					
**Normal sperm morphology**	31.5	±13.0	49.1	±12.8	**4 × 10^−4^**
(%)					
**Round cells**	0.2	±0.4	0.3	±0.6	0.70
(10^6^ round cells/mL)					
**Leukocytospermia**	0.0	±0.0	0.1	±0.4	0.09
(10^6^ leukocytes/mL)					
**Oligozoospermia**	30	100	0	0.0	
(*n*, %)					
**Asthenozoospermia**	11	36.7	0	0.0	
(*n*, %)					
**Teratozoospermia**	10	33.3	0	0.0	
(*n*, %)					

s.d: standard deviation; *n*: number; %: percent.

**Table 3:** Sperm nuclear alterations observed in case and control groups.

Sperm Nuclear Alterations	Case Group		Control Group		*p*
	mean ± s.d	Spz Number	mean ± s.d	Spz Number	
**Cytoplasmic ROS**	24.3 ± 20.7	507 ± 151	9.4 ± 7.4	540 ± 133	**0.039**
(%)					
**Nuclear ROS**	30.3 ± 20.3	507 ± 151	26.1 ± 13.3	540 ± 133	0.66
(%)					
**8-OHdG** **positive spz**	3.9 ± 2.8	536 ± 46	3.0 ± 2.3	542 ± 72	0.7
(%)					
**DNA** **fragmentation**	9.8 ± 6.4	569 ± 151	7.1 ± 5.3	591 ± 164	0.49
(%)					
**Aneuploidy**	0.3 ± 0.2	5277 ± 74	0.4 ± 0.2	5249 ± 142	0.28
(%)					
**Diploidy**	0.3 ± 0.2	5277 ± 74	0.3 ± 0.2	5249 ± 142	0.99
(%)					
**Total** **Chromosome** **abnormalities**	0.7 ± 0.3	5277 ± 74	0.8 ± 0.4	5249 ± 142	0.7
(%)					
**Abnormal** **chromatin** **condensation**	15.2 ± 8.2	542 ± 110	9.3 ± 4.2	624 ± 114	**0.006**
(%)					
**Mean number** **of telomeres**	21.7 ± 4.3	200 ± 18	18.8 ± 3.0	214 ± 15	**0.049**
(Fluorescent signals per spz)					
**Relative** **Telomere** **length**	59.6 ± 30.7	200 ± 18	65.8 ± 26.5	214 ± 15	0.7
(FRU)					

8-OHdG: 8-oxo-deoxyguanosine; FRU: fluorescence relative units; ROS: reactive oxygen species; s.d.: standard deviation; spz: spermatozoa; %: percent.

## Data Availability

All data that support the findings of this study are available from the corresponding author upon request.

## Supplementary Materials

The following are available online at https://www.mdpi.com/article/10.3390/antiox10040593/s1, Table S1: Semen parameters in control group for males who achieved pregnancy before sperm sampling.
