# Supplementary material for: Oxidative Stress Is Associated with Telomere Interaction Impairment and Chromatin Condensation Defects in Spermatozoa of Infertile Males
Source: Antioxidants (Basel). 2021 Apr 12;10(4):593. doi: 10.3390/antiox10040593 (PMC8069055; doi:10.3390/antiox10040593)
Supplement: Supplementary file 1 [file antioxidants-10-00593-s001.pdf]

Table S1. Semen parameters in control group for males who achieved pregnancy before sperm sampling.

Semen Parameters	Control Group			
	With Child n = 12		Without Child n = 10	
	mean ± s.d			
**Sexual abstinence** (days)	**5.7**	**± 2.0**	5.4	±2.3
**Volume** (mL)	4.5	± 2.4	4.2	±1.1
**Concentration** (10^6/mL)	51.4	± 24.1	43.2	±15.7
**Total sperm number** (10^6/ejaculate)	214.1	± 124.4	177.9	±68.1
**Sperm progressive motility** (a + b, %)	39.2	± 5.2	42.0	±5.9
**Vitality** (live spermatozoa, %)	80.4	± 6.2	79.0	±7.0
**Normal sperm morphology** (%)	47.2	± 14.6	51.3	±10.9
**Round cells** (10^6 round cells/mL)	0.3	± 0.5	0.3	±0.7
**Leukocytospermia** (10^6 leukocytes/mL)	0.05	± 0.1	0.2	±0.5
**Cytoplasmic ROS** (%)	10.6	± 7.7	8.0	±7.2
**Nuclear ROS** (%)	25.3	± 13.0	27.7	±14.2
**8-OHdG positive spz** (%)	3.3	± 2.6	2.6	±1.9
**DNA fragmentation** (%)	8.1	± 6.3	6.0	±4.0
**Total chromosome abnormalities** (%)	0.85	± 0.5	0.7	±0.3
**Abnormal chromatin condensation** (%)	10.2	± 4.8	8.3	±3.2
**Mean number of telomeres** (fluorescent signals per spz)	19.0	± 2.8	18.6	±3.4
**Relative telomere length** (FRU)	57.3	± 13.7	59.2	±30.4

8-OHdG: 8-Oxo-deoxyGuanosine; FRU: fluorescence relative units; *n*: number; ROS: reactive oxygen species; s.d: standard deviation; spz: spermatozoa; %: percent.
